# Epigenetic Regulation of β Cell Identity and Dysfunction

**DOI:** 10.3389/fendo.2021.725131

**Published:** 2021-09-24

**Authors:** Xiaoqiang Sun, Liu Wang, S. M. Bukola Obayomi, Zong Wei

**Affiliations:** ^1^ Department of Physiology and Biomedical Engineering, Mayo Clinic Arizona, Scottsdale, AZ, United States; ^2^ Tianjin Fourth Central Hospital, Tianjin, China; ^3^ The Fourth Central Hospital Affiliated to Nankai University, Tianjin, China; ^4^ The Fourth Central Hospital Clinical College, Tianjin Medical University, Tianjin, China

**Keywords:** epigenetic, beta cell dysfunction, histone acetylation, non-coding RNAs, DNA methylation, chromatin accessibility

## Abstract

β cell dysfunction and failure are driving forces of type 2 diabetes mellitus (T2DM) pathogenesis. Investigating the underlying mechanisms of β cell dysfunction may provide novel targets for the development of next generation therapy for T2DM. Epigenetics is the study of gene expression changes that do not involve DNA sequence changes, including DNA methylation, histone modification, and non-coding RNAs. Specific epigenetic signatures at all levels, including DNA methylation, chromatin accessibility, histone modification, and non-coding RNA, define β cell identity during embryonic development, postnatal maturation, and maintain β cell function at homeostatic states. During progression of T2DM, overnutrition, inflammation, and other types of stress collaboratively disrupt the homeostatic epigenetic signatures in β cells. Dysregulated epigenetic signatures, and the associating transcriptional outputs, lead to the dysfunction and eventual loss of β cells. In this review, we will summarize recent discoveries of the establishment and disruption of β cell-specific epigenetic signatures, and discuss the potential implication in therapeutic development.

## Introduction

Diabetes is a chronic metabolic disease characterized by elevated blood glucose levels and inducing serious damage in macrovascular and microvascular systems. According to the International Diabetes Federation (IDF) Diabetes Atlas (9th edition), worldwide population of diabetes mellitus patients reached 463 million in 2019, and is estimated to reach 578 million by 2030, and 700 million by 2045 (1). In the past three decades, the prevalence of Type 2 Diabetes Mellitus (T2DM), the most prevailing form (90-95%) of diabetes, has risen dramatically (1).

T2DM is characterized by insulin resistance and impaired insulin secretion from pancreatic β cells. In the past decade, β-cell dysfunction has been recognized as a driving force in T2DM (2). T2DM has a strong genetic component and is the result of the complex interplay between genes and the environment. Remarkably, the concordance rate of T2DM in monozygotic twins is about 70%, while the concordance in dizygotic twins is only about 20%–30% (3). In the past decades, genome-wide association studies (GWAS) has established linkage between T2DM and multiple genes regulating β-cell proliferation (4) and β-cell lineage determination (5) (e.g., Pdx1, Nkx6.1, and Mafa). However, the identified coding region SNPs are still insufficient to explain the heritability of T2DM (6, 7), as the majority of SNPs identified in GWAS studies are located in non-coding regions (8). A series of recent studies indicated that the GWAS identified SNPs are enriched in the β cell-specific cis-regulatory regions (9–11). In this framework, understanding the epigenetic dynamics of the regulatory regions in homeostatic and dysfunctional β-cells may offer insights into the interplay between environmental parameters and the genetics of T2DM. This review will focus on the biological function of epigenetic regulation including: chromatin accessibility, histone modifications, DNA methylation, and non-coding RNAs in human and rodent pancreatic β cell homeostasis and pathogenesis.

## The Major Classes of Epigenetic Modification and Their Roles in Regulating β Cell Dysfunction

### Chromatin Modifications and Accessibility

In eukaryotes, histones and DNA assemble into nucleosomes. Due to the difference in amino acid composition and molecular weight, histones are mainly divided into 5 categories: H1, H2A, H2B, H3 and H4. Except for H1, the other four histones all combine in the form of dimers to form the core of nucleosomes. DNA is wrapped around the core of the nucleosome. H1 binds to the linker DNA between the nucleosomes. N-terminal tails of histones are subject to various post-translational modifications including: acetylation, methylation, and ubiquitinoylation of lysine (K), methylation of arginine (R), and phosphorylation of serine (S) and threonine (T) (12), that can impact gene expression by altering chromatin structure or recruiting histone modifiers. The state of the chromatin structure is regulated by histone-modifying enzymes, which chemically change histone tail residues (13).

Histone acetylation is one of the extensively studied histone modifications in β-cell adaptation (14). The acetylation on lysine is modified, or removed, by histone acetyltransferase (HAT) and histone deacetylases (HDAC). Acetylation of lysine on histone tails removes the positive charge on the histones to weaken the interaction between the positively-charged histones and the negatively-charged DNA. The lysine acetylation also creates docking sites for reader proteins in addition to increased accessibility of chromatin for transcription factor (TF) binding resulting in downstream gene expression changes (13). The acetylation of histones have been associated with islet development, function, and the development of T2DM. Histone H3 lysine 27 acetylation (H3K27ac), a mark associated with active enhancers, is decreased in both 2-week and 10-week intrauterine growth retardation (IUGR) rats which is associated with increased risk of adulthood T2DM (15). The association between H3K27Ac, gene expression changes, and functional defects in pancreatic islets have been described in high fat diet induced obese mice (16). To establish the causality between histone acetylation and islet function, many studies have utilized the comprehensive collection of HDAC inhibitors. Haumaitre et al. (17, 18) studied the role of HDAC in rat embryonic pancreatic development, and found that HDAC inhibitors reduce the differentiation of pancreatic exocrine cells and increase Neurogenin 3 positive (Ngn3^+^) endocrine progenitor cells. These studies demonstrated that the dynamic changes of histone acetylation have a crucial effect on the final number of different types of endocrine cells (i.e.: α, β, δ, ϵ, PP cells). In addition to lineage specification, a series of studies [reviewed in (19)] also showed that HDACs are crucial in diabetes pathogenesis, as selective HDAC inhibitors are able to increase the insulin secretion function of β-cells, or expansion of β cell mass (20, 21). Pan class I HDAC (HDACs located exclusively in the nucleus) inhibitors, such as vorinostat (SAHA), and TSA, have been reported to reduce β cell apoptosis under inflammatory stress, and increase insulin secretion in β-cells. Mechanistically, the genomic and proteomic data demonstrate that SAHA robustly increases a low-abundance histone 4 polyacetylation state, which serves as a preferred binding substrate for several bromodomain-containing proteins (22). Unfortunately, the toxicity and growth inhibition of the pan HDAC inhibitors may overweigh their benefits on islets. Out of all class I HDACs, HDAC3 has been a particularly promising target for β cell function. The genetic deletion of HDAC3 (23) as well as a specific HDAC3 inhibitor BRD3308 (21), consistently showed an improvement of β cell function through enhanced insulin secretion. Besides class I HDACs, the class II HDACs, which are located in both the cytoplasm and nucleus, are also involved in β cell function. The genetic deletion of different members of class II HDACs (HDAC4/5/9) leads to the increase of β cells and/or δ cells, and its overexpression decreases the number of β and δ cells (24). Another class II HDAC, HDAC6, was also found to be downregulated in db/db mouse islets (25). However, unlike HDAC3, the underlying mechanisms of class II HDACs in regulating β cells epigenetic signatures remains unclear.

The specificity of lysine acetylation regulation is achieved by a collaborative effort of HAT/HDACs and their TF binding partners. For example, it was reported that pancreatic and duodenal homeobox 1 (PDX1) regulates the acetylation of histones (26). At high glucose levels, PDX1 forms a complex with histone acetyltransferase p300, forms open chromatin at the insulin promoter, then stimulates insulin expression. At low glucose concentrations, PDX1 recruits HDAC1 and HDAC2 to inhibit insulin gene expression (27). It is likely that p300 serve as docking sites for other TFs and coregulators to enhance gene transcription in β-cells (28). It has been reported that glucose stimulates the recruitment of p300 to the promoter region of the pro-apoptotic factor Txnip gene in human islets (29). Consistent with this model, a recent study reported that the proteasomal degradation of p300 leads to beta-cell apoptosis (30). Ruiz et al. predicts that knock-down/inhibition of p300 under normal conditions blocks not only the expression of proapoptotic factors such as Txnip, but also the expression of survival factors which overall favors the emergence of beta-cell apoptosis (30).

Besides class I and II HDACs, other lysine deacetylases, such as the Sirtuin family members, also contribute to β cell dysfunction. Sirtuin 1(SIRT1), a nicotinamide adenosine dinucleotide (NAD+)-dependent deacetylase, is able to deacetylate both histones and certain TFs to regulate the expression of genes (31–33). In β cells, SIRT1 enhances glucose-dependent insulin secretion (34, 35), while its expression is disrupted under inflammatory stress (36).

Another extensively studied modification of histones is the methylation on lysine and arginine. Lysine can undergo mono-, di- and tri-methylation respectively, while arginine can only undergo mono- and di-methylation. There are 5 lysine sites on Histone H3 that can be methylated. Given the significance of histone methylation in regulating gene expression, it comes as no surprise that disruption of histone methylation leads to dysfunctional β cells. Trimethylation of histone H3K4 is mainly concentrated in the promoter region of active transcription. A recent study found that disruption of H3K4Me, by deletion of Set7/9, reduces β-cell essential genes such as Pdx1, and compromises the islet GSIS function (37, 38). Besides lysine, the methylation of arginine has also been linked to β cell function. Recently, Jian and colleague (39) found that an islet-specific knock out of *PRMT5*, an arginine methyltransferase for H3R8 di-methylation, led to reduced insulin secretion, and impaired glucose tolerance and glucose-stimulated insulin secretion (GSIS). Using a genetic mouse model to impair H3K4 methyltransferase activity of TrxG complexes, Vanderkruk et al. find that reduction of H3K4 methylation significantly reduces insulin production and glucose-responsiveness and increases transcriptional entropy, indicative of a loss of β-cell maturity. The data implicate H3K4 methylation dysregulation as destabilizing β-cell gene expression and contributing to cell dysfunction in T2D (40).

Chromatin accessibility enables TF binding and therefore is tightly associated with gene regulation. Chromatin accessibility is regulated by a number of chromatin remodeling complexes, such as the ATP-dependent Switch/sucrose non-fermentable (SWI/SNF) complex (41). Though known to be essential for general transcription, recent studies have started to reveal the distinct function of individual components of SWI/SNF complex in regulating β cell function. The ATPase of the SWI/SNF complex, BRG1, was shown to be recruited by PDX1 and maintains the PDX1 targets expression at high glucose levels, as well as progenitor differentiation (42, 43). In contrast, BRM, another ATPase of the SWI/SNF complex, represses the PDX1 target genes expression at low glucose concentrations (42). Our previous study found that vitamin D switches the binding partner of vitamin D receptor (VDR) from the BRD9-containing SWI/SNF complex to the BRD7 containing PBAF complex, resulting in increased chromatin accessibility at VDR targets and enhancing the anti-inflammatory and anti-stress responses in β-cells (44). Another recent study showed that deletion of ARID1A-containing BAF complex in β cells promotes β regeneration under an STZ challenge (45). Therefore, the various compositions of the SWI/SNF complex defines the diverse molecular and biological function of chromatin remodeling complexes in β cells. The underlying regulatory mechanisms of these complexes, both antagonistic and collaborative in a context-dependent fashion, remain to be characterized.

One essential goal of the chromatin study is to define the regulome of β cells in homeostasis and diabetes. A number of pioneer studies has defined active and repressive cis-elements by histone marks in progenitor and mature β cells (46–49). Importantly, the islet enhancers featured by the active histone markers (H3K4Me1/2 and H3K27Ac) contain many T2D susceptibility SNPs (47). Stretch enhancers are cell type specific and are associated with increased expression of genes involved in cell-specific processes. Parker et al. find that genetic variations associated with common disease are highly enriched in stretch enhancers; stretch enhancers specific to pancreatic islets harbor variants linked to type 2 diabetes and related traits (50). Arda et al. identify and characterize chromatin features underlying cell-type-specific gene expression in human pancreatic α, b, duct, and acinar cells (51). The work prioritizes candidate risk genes for pancreatic diseases such as diabetes mellitus (51). Technological advances, such as ATAC-seq (52)and DNase-seq (53), have enabled mapping open chromatin with much higher resolution. Importantly, recent developments in single-cell approaches, such as single cell RNA-seq and single cell ATAC-Seq has enabled identification of the epigenome or transcriptome of a single cell resolution, providing a high-resolution view of cell-to-cell variation (54, 55). Utilizing these technologies, a recent study showed that T2DM GWAS SNPs are significantly enriched in β cell-specific and ubiquitous open chromatin regions, but not in alpha or delta cell-specific open chromatin (56). Khetan et al. profiled chromatin accessibility in pancreatic islet samples from 19 genotyped individuals and identified 2,949 SNPs associated with *in vivo* cis-regulatory element use. The islet caQTL analysis nominated putative causal SNPs in 13 T2D-associated GWAS loci, linking 7 and 6 T2D risk alleles, respectively, to gain or loss of *in vivo* chromatin accessibility (57). Using scATAC-seq, Chiou et al. profiled 15,298 islet cells and identified 12 clusters, including a, β and d cell signatures (9). By defining the co-accessibility of regulatory elements across single cells, the authors were able to link regulatory variants to putative target genes. An example is the co-accessibility between *KCNQ1* intron 3, which harbors a T2D variant rs231361, and the distal INS promoter (9). Genome editing of this SNP resulted in changes of INS expression, suggesting a causality between the KCNQ1 SNPs and the distal INS gene expression (9). Lu et al. provide evidence of chromatin dysregulation in type 2 diabetes in mice and humans. Loss of Polycomb silencing in mouse pancreas triggers hyperglycemia-independent dedifferentiation of β cells and diabetes, suggesting a two-hit (chromatin and hyperglycemia) model for loss of β cell identity in diabetes (58). Future studies connecting single cell transcriptome/epigenome with spatial profiling will significantly enhance our understanding of islet structure and function.

### DNA Methylation

DNA methylation is an epigenetic mechanism by which methyl groups are transferred from S-adenosylmethionine to the C5 position of the cytosine to form 5-methylcytosine *via* DNA methyltransferases (DNMT). In mammals, DNA methylation is mostly found in CpG dinucleotides (59). Most CpG islands located in the promoter region of active genes are unmethylated, whereas hypermethylation of CpG islands are associated with gene silencing (60). Transcription silencing caused by DNA methylation is achieved either directly by inhibiting the binding of transcription factors to DNA, or indirectly by recruiting other chromatin-modifying proteins, such as DNA methylation binding proteins and histone deacetylation HDAC (61). As an epigenetic modification, DNA methylation plays an important role in the regulation of chromatin structure and gene expression as well as participating in a variety of biological processes, including β cell differentiation, homeostasis, and diabetes pathogenesis. A study using whole-genome bisulfite sequencing on human islets from patients with T2DM and healthy controls identified 25,820 T2DM differentially methylated regions (DMRs), with 12,124 DMRs reduced and 13,696 DMRs elevated in T2DM islets (62). In addition, 457 genes associating DMRs are differentially expressed in T2DM islets (62). In another genome-wide DNA methylation profiling using human pancreatic islets, 1,649 CpG sites and 853 genes with differential DNA methylation in pancreatic islets from type 2 diabetic donors were identified (63). Functional analysis showed that these genes can directly affect insulin secretion in pancreatic β cells (63). Long-term exposure to elevated free fatty acids (FFA) and glucose levels impair islet insulin secretion (64). Elin et al. (65) found that glucolipotoxicity impaired insulin secretion and changed the expression of 1,855 genes, including 35 of 264 T2DM candidate genes identified by genome-wide association studies such as *TCF7L2*, *BCL11A*, and *CDKN2B*, and the genes of downregulated metabolic pathways were enriched. Importantly, 1469 differentially expressed genes also had DNA methylation altered (e.g., *CDK1, FICD, TPX2*, and *TYMS*) (65). This data suggests that changes in DNA methylation affected the insulin secretion and played a key role in β cell dysfunction in T2DM. Besides insulin secretion, DNA methylation is also associated with cell fate decision since the loss of DNA methylation promotes pancreatic α-cell to β-cell transdifferentiation (66, 67).

Cytokine stress causes β cell dysfunction. It was demonstrated that interleukin1‐β (IL‐1β) induced aberrant DNA methylation to cause β-cells dysfunction (68). After treated with IL‐1β, the expression level of Calcium/calmodulin‐dependent serine protein kinase (CASK) declined to nearly 40%, while methylation at the Cask promoter increased. After treatment with DNA methyltransferase (DNMT) inhibitor, 5‐Aza‐2’‐deoxycytidine (5‐Aza‐dC), or DNMTs siRNAs, CASK expression and insulin secretion were partially rescued.

In addition, DNA methylation mediates intergenerational epigenetic effects. Elmar et al. performed a genome-scale analysis of differential DNA methylation in whole blood after periconceptional exposure to famine during the Dutch Hunger Winter (69). They found 181 regions as prenatal-malnutrition associated with differentially methylated regions (69). Zahra and colleague (70) found a link between pancreatic β-cell dysfunction and hypomethylation of the CDKN2A/B promoter in offspring of, streptozotocin-induced Gestational diabetes mellitus (GDM), rats. Although the associations between epigenetic inheritance and methylation changes have been identified, it remains unknown that these epigenetic changes are causative to diabetes progression of β cell dysfunction. Also, a contradictive study did not find any association between DNA methylation changes in sperm and the epigenetic inheritance of diet-induced phenotypes, and also there were not any DNA methylome changes in offspring after utero caloric restriction (71). Therefore, future studies of DNA methylation in inter-generational inheritance, using better defined models and robust statistics, are needed.

DNA methylation of regulatory elements is dynamic with maturation and aging of pancreatic β cells. β cell function is improved during the maturation process, as predicted by methylome and transcriptome changes [see review (72)]. Interestingly, Avrahami et al. demonstrated that in rodents, DNA methylation changes are associated with aging-induced β-cell function improvement (73). Using genome-wide DNA methylome analysis in purified β-cells from young (4-6 weeks) and old mice (16-20 months), they found that promoters of proliferation genes (e.g., Ki67, Plk1, and Ccnd3) are hypermethylated in aged mice, while enhancers of genes responsible for β-cell function (e.g., Foxa2, Nkx6.1, and Neurod1) are hypomethylated, and correlated with the increase of gene expression and functional improvement in aged mice (73). Whether these methylation dynamics and its corresponding gene expression changes are conserved in aged human islets remains to be characterized.

### Non-Coding RNA

Non-coding RNA (ncRNA) refers to functional RNA that does not encode protein (74). ncRNAs are essential in the regulation of epigenetics and are widely involved in the regulation of embryonic development, cell fate determination, and material metabolism (74). In recent years, mounting evidence suggests that the development and differentiation of β cells, the synthesis and secretion of insulin and the development of T2DM are not only precisely regulated by transcription factor, but also mediated by ncRNAs (14, 75, 76).

One of the most prominent and widely studied class of ncRNAs is miRNA, which have been used as biomarkers in metabolic diseases (77, 78). Profiling of miRNAs in blood and pancreatic islets indicates that various miRNAs exert essential functions in the epigenetic regulation network in the progression of T2DM (79). A number of recent studies, focusing on the regulatory machinery of miRNA processing, demonstrated that miRNA plays an important role in β-cell proliferation and survival. Dicer is the key enzyme for miRNA cleavage and maturation. In Pdx1-Cre mediated, pancreas specific knock-out of Dicer1 mice, the pancreatic development was impaired and the β cell mass reduced, which lead to embryonic lethality. Loss of Dicer also reduced the Ngn3^+^, β cell progenitor population, leading to impaired endocrine cell development. In endocrine progenitor cells, loss of Dicer lead to morphological defects, reduced insulin expression, and hyperglycemia in neonatal mice. Together these loss-of-function studies indicated that the whole class of miRNA is necessary for pancreatic development and differentiation of pancreatic endocrine cells (80, 81).

**Figure 1:**
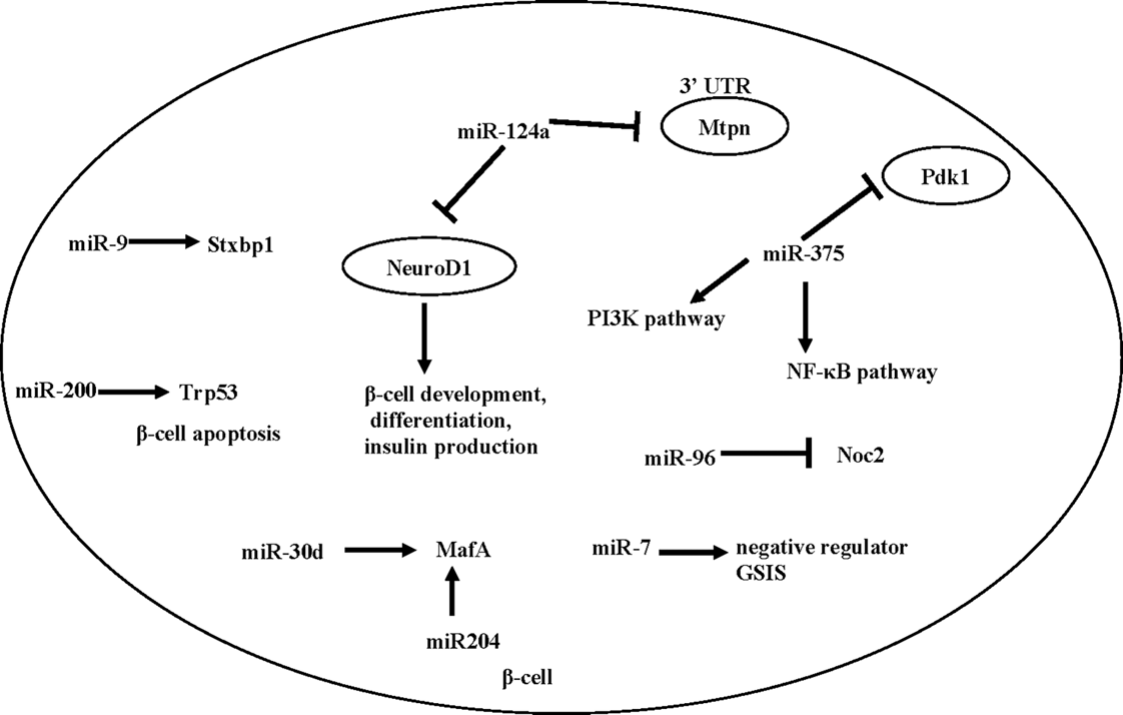
miRNAs involved in insulin release in pancreatic beta cells and beta cell fate.

A number of individual miRNAs have been reported to mediate β -cell development, function, or T2D development. Here we summarize a number of miRNAs that have been associated with β cell function (**Figure 1**).

miR-375 is an important miRNA in the differentiation process of β cells. Kloosterman et al. (82) found that inhibition of miR-375 can lead to disrupted development of the embryonic pancreas in zebrafish, and in particular, a scattered distribution of pancreatic islets. Knockout of miR-375 caused abnormal pancreatic islet morphology in mice, reduced total amount of α and β cells, reduced insulin secretion and increased blood glucose (82). miR-375 knockout mice showed reduced β cell mass, together with an increase in plasma glucagon and gluconeogenesis, suggesting that miR-375 levels are essential for glucose homeostasis and proliferation of β-cells (83), though its direct effect on β cell remains to be determined.

miR-7 is expressed in the embryonic and adult pancreas. An early study by Margarita et al. (84) found that inhibition of miR-7 during early embryonic development lead to reduced β cell mass, decreased insulin secretion, and impaired glucose tolerance in mice after birth. Interestingly, Latreille et al. (85), using β cell specific KO of miR-7 mice, showed that miR-7 is a negative regulator of GSIS in β cells. This discrepancy may be caused by the different genetic model (antisense *vs*. β cell specific knock-out). Future studies will be needed to characterize the potentially diverse function of miR-7 in β cells and other metabolic tissues.

miR-200 is another essential player in β cell health. Bengt-Frederik et al. (86) found that overexpressing miR-200 induces β cell apoptosis by suppressing the antiapoptotic and stress-resistance pathways, whereas deletion of miR-200 ameliorated diabetes progression. Mechanistically, miR-200 activates Trp53 and promotes β cell apoptosis (86).

Besides β cell development and survival, a number of studies also showed that islet-enriched miRNAs influence insulin transcription or secretion [reviewed in (87)]. Specifically, Tang et al. (88) found that the expression of miR-375, miR-124a, miR-107, miR-30d, miR-690 and miR-let7 were significantly up-regulated when exposed to high glucose, while the expression of miR-296, miR-484 and miR-690 were down-regulated. Importantly, when miR-30d is overexpressed, the transcription of insulin increases (88), possibly caused by an increased level of *MafA* (89). In addition, miR-204 can also directly target and regulate *MafA* (90). Besides miR-30d, other miRNAs involved in insulin expression include miR-24, miR-26, miR-182, and miR-148, as depletion of these miRNAs in cultured β cells or mouse islets reduced the activity of insulin gene promoters and led to a decrease in insulin content (91). In contrast, overexpression of miR-375, an islet-specific miRNA, inhibits insulin secretion and has a negative regulatory effect on glucose-stimulated insulin expression and/or β cell proliferation, possibly through the NF-κB pathway (92) or the PI3K pathway (93, 94).

Besides insulin synthesis, several microRNAs, including miR124a, miR-9 and miR-96, are found to regulate exocytosis and insulin secretion (95). Both miR124a and miR-96 are negative regulators for insulin secretion in Min-6 cells, by down-regulating exocytosis genes such as Rab27a and Noc2 (95, 96). miR-9 is a negative regulator of β-cell secretion function by regulating transcription of Stxbp1 (97). A number of miRNAs also directly regulate insulin secretion by targeting GLP-1 signaling. An example is miR-204, which represses expression of the GLP-1 receptor in INS1 cells, as well as in mouse and human islets (98).

In summary, miRNAs are essential players in β-cell differentiation and function. Future studies using genomics and system biology approaches may reveal a more comprehensive and quantitative landscape of the miRNA regulatory network in β cells.

LncRNAs can be located in the nucleus or cytoplasm and regulate the expression of protein-coding genes during transcriptional and post-transcriptional stages (99). Several recent studies suggest that lncRNAs are crucial components of the islet regulome and may have a role in diabetes pathogenesis (100). Morán et al. identified multiple islet-specific lncRNAs that are dynamically regulated in human β cells and showed that they are an integral component of β cell differentiation and maturation (101). In particular, depletion of HI-LNC25, a β cell-specific lncRNA, downregulated GLIS3 mRNA (101), suggesting that islet lncRNAs can directly control transcription (101). Another lncRNA involved in β cell transcriptome is MALAT1, which was first described as a highly expressed nuclear lncRNA regulating alternative splicing (102). MALAT1 has been reported to modulate the active histone marks of the Pdx1 promoter in MIN6 cells (103) and to promote the stability of the polypyrimidine tract binding protein 1 (Ptbp1) in the nucleus. A number of lncRNA have also been associated with insulin secretion and β cell responses to nutrients. The lncRNA Maternally expressed gene 3 (Meg3) has been shown to regulate insulin secretion (104). Kong et al. identified ten different lncRNAs that were differentially expressed in the INS-1 cells after treated with a combination of high glucose and palmitate acid (105). Some lncRNAs are located in proximity of β-cell essential TFs, suggesting a cis-regulatory mechanism. For example, the lncRNA PLUTO, transcribed antisense to Pdx1, modulates the expression of Pdx1 by promoting the looping of an enhancer within the Pdx1 promoter (106). Silencing of PLUTO in a human β cell line, EndoC-βH3, impaired GSIS (106). Lastly, it should be noted that besides β cells, lncRNA in other metabolic tissues are also able to regulate glucose metabolisms and diabetes progression. For example, GWAS studies showed that misexpression of several lncRNAs are correlated to diabetes complications (107, 108). The role of lncRNAs in peripheral metabolic tissues, especially in energy homeostasis, has also been explored (90).

In 2012, more than 1,000 conserved lncRNAs were found in mouse and human pancreatic islets (101). With the large number of identified islet-specific lncRNAs, the functional characterization of lncRNAs remains challenging. The most commonly used strategy is loss-of-function assays by RNA interference. For example, knockdown of the lncRNA blinc1 in MIN6 cells demonstrated that blinc1 is a novel cis-regulator of the islet transcription factor Nkx2-2 (109). additional methods to characterize the molecular activity of a lncRNA include identifying its physically interacting partners, with either a protein-centric or an RNA-centric approach. Protein-centric methods such as RNA immunoprecipitation (110) or cross-linking immunoprecipitation (111), use antibodies to immunoprecipitate RNA binding protein complexes from the cell lysate. Some newer versions of these techniques, such as Capture Hybridization Analysis of RNA Targets (CHART) (112), Chromatin Isolation by RNA Purification (ChIRP) (113), and RNA Antisense Purification (RAP) (114), are also available. As the tools for studying lncRNA function continue to expand, future studies will continue to characterize the versatile function of lncRNAs in regulating β-cell biology and diabetes pathophysiology.

## Conclusion

The essential role of epigenetics in the development and function of pancreatic β-cells, as well as the dysfunction and β cell failure in T2DM, is supported by the increasing number of studies in humans and model organisms. Numerous studies over the past two decades have established the a few fundamental principles of epigenetic regulation in diabetes: 1) it is now clear that the combination of chromatin modification, DNA methylation, and non-coding RNA together define the identity of β cells; 2) the dysregulation of β cell epigenetic signatures is a key event in the pathogenesis of diabetes; and 3) the molecular machinery that define these epigenetic signatures can be potential targets in the therapeutic development. Future research will be needed to specifically define the epigenetic events regulating specific cellular function of β cells, such as insulin secretion, regeneration, and immune tolerance, etc. Understanding the epigenetic regulation mechanism in these processes is of great significance for developing next generation treatment for T2DM.

## Author Contributions

XS and LW performed the systematic literature search and edited the manuscript. SO reviewed and edited the manuscript. ZW supervised and edited the manuscript. All authors contributed to the article and approved the submitted version.

## Funding

This project was supported by a grant from NIH DK120808 (ZW), Roubos Family Fund in research (ZW), and a fellowship from Tianjin Fourth Central Hospital (XS).

## Conflict of Interest

The authors declare that the research was conducted in the absence of any commercial or financial relationships that could be construed as a potential conflict of interest.

## Publisher’s Note

All claims expressed in this article are solely those of the authors and do not necessarily represent those of their affiliated organizations, or those of the publisher, the editors and the reviewers. Any product that may be evaluated in this article, or claim that may be made by its manufacturer, is not guaranteed or endorsed by the publisher.
